# Nutraceuticals and Physical Activity as Antidepressants: The Central Role of the Gut Microbiota

**DOI:** 10.3390/antiox11020236

**Published:** 2022-01-26

**Authors:** Sabrina Donati Zeppa, Fabio Ferrini, Deborah Agostini, Stefano Amatori, Elena Barbieri, Giovanni Piccoli, Piero Sestili, Vilberto Stocchi

**Affiliations:** 1 Department of Biomolecular Sciences, University of Urbino Carlo Bo, 61029 Urbino, Italy; sabrina.zeppa@uniurb.it (S.D.Z.); f.ferrini2@campus.uniurb.it (F.F.); deborah.agostini@uniurb.it (D.A.); elena.barbieri@uniurb.it (E.B.); giovanni.piccoli@uniurb.it (G.P.); piero.sestili@uniurb.it (P.S.); 2 Università Telematica San Raffaele, 00166 Rome, Italy; vilberto.stocchi@uniroma5.it

**Keywords:** depression, exercise, gut–brain axis, gut microbiota, nutraceuticals

## Abstract

Major depressive disorder (MDD) is a common mental illness. Evidence suggests that the gut microbiota play an essential role in regulating brain functions and the pathogenesis of neuropsychiatric diseases, including MDD. There are numerous mechanisms through which the gut microbiota and brain can exchange information in a continuous, bidirectional communication. Current research emphasizes the interexchange of signals influenced by the gut microbiota that are detected and transduced in information from the gut to the nervous system involving neural, endocrine, and inflammatory mechanisms, suggesting a relationship between oxidative stress and the pathophysiology of MDD via the hyperactivation of inflammatory responses. Potential sources of inflammation in the plasma and hippocampus of depressed individuals could stem from increases in intestinal permeability. Some nutraceuticals, such as specific probiotics, namely psychobiotics, polyphenols, carotenoids, butyrate, and prebiotics, have been demonstrated to exert an antidepressant activity, but most of them need to be metabolized and activated by gut microorganisms. By inducing changes in the gut microbiota composition, physical exercise might also exert a role in alleviating depression-like symptoms. The mutual relationships among nutraceuticals, exercise, and depression will be discussed, and the potential role of the gut microbiota as a therapeutic target to treat depression will be explored.

## 1. Introduction

Major depressive disorder (MDD) is a common psychiatric illness characterized by feelings of guilt, low mood, and cognitive deficits, as well as physical changes, such as weight loss, disturbed sleep, and appetite [1]. More than 350 million people have been affected by this disorder, and significantly greater depression has been observed during the pandemic than at any other time during the last few years [2].

The molecular basis and diagnostic biomarkers of MDD are still critical, although several hypotheses have been formulated to explain its pathophysiological mechanisms.

A relationship between oxidative stress and the pathophysiology of MDD has been demonstrated. The reactive oxygen species (ROS) function as second messengers of the immune system, and increased levels can induce the hyperactivation of inflammatory responses. In particular, neuroinflammation and brain cell damage, leading to cognitive impairment, can be induced by the excessive production of ROS and reactive nitrogen species (RNS) [3,4,5].

Furthermore, an increase in pro-inflammatory cytokines in depressive patients, including tumor necrosis factor-alpha (TNF-α), has been identified. TNF-α is known to induce apoptosis, disorders in synaptic plasticity, and depressive behaviors [6].

Recently, some evidence suggests that the gut microbiota play an essential role in the function of the central nervous system (CNS) and the pathophysiology of several mental diseases, including depression [7]. Preclinical studies revealed that microbiota seem to occupy a central role in the functioning of multiple gut–brain communications [8], such as with the autonomic nervous system, the enteric nervous system, the neuroendocrine system, and the immune system.

Conventional therapies are antidepressant drugs that increase the synaptic concentration of monoamines. However, antidepressant therapies often are not effective, suggesting that other mechanisms rather than monoamines deficiency are involved in the pathophysiology of depression. The new classes of antidepressants still have antimicrobial effects, and on the other hand, β-lactams and tetracyclines also have potential antidepressant properties [9,10]. Both antidepressants and antimicrobials present neuroprotective/antidepressant and antimicrobial effects. This evidence supports the hypothesis of a close functional interaction between gut microorganisms and the CNS.

Moreover, gut microbiota imbalance has been observed in people affected by MDD [11]. In this light, recently, it has been demonstrated that “psychobiotics”, a new family of probiotics, can influence gut–brain relationships [12]. Beneficial psychotropic effects of *Lactobacillus plantarum* ATCC 8014 accompanied with inulin have been reported by Morshedi et al. [12]. Other nutraceuticals such as polyphenols, carotenoids, and butyrate have also demonstrated an antidepressant activity, but most of them need to be metabolized and activated by gut microorganisms.

Finally, the possible mechanisms of action of physical exercise in the management of depressive symptoms through the microbiota–gut–brain axis will be debated. Exercise has been shown to increase microbiota diversity and richness, consequently improving the metabolic profile and immunological responses [13]. Exercise-induced modifications of specific microbiota taxa could alleviate stress and depression-like symptoms [14].

The mutual relationships among nutraceuticals, gut microbiota, exercise, and depression will be discussed, and in perspective, the potential role of the intestinal microbiota as a therapeutic target to treat depression will be explored.

## 2. Microbiota–Gut–Brain Axis

The human gut microbiota contains a complex ecosystem, and it has been recognized as an essential metabolic organ with at least 1800 bacterial genera and over 40,000 species of microorganisms [15]. Despite the presence of many phyla of bacteria in the gut, the most prominent are the *Firmicutes* (including *Lactobacillus*, *Clostridium*, and *Enterococcus* genus) and *Bacteroidetes* (e.g., *Bacteroides* genus) [16]. The gut microbiota composition is variable among individuals, and in addition, it is extremely dynamic, influenced by factors such as genetics, age, diet, metabolism, geography, and stress [17]. Recently it has been suggested that the gut microbiota could result from the genetic and environmental determinants of a subject, and it could be a determinant of future diseases and outcomes to therapeutical treatments [17]. The richness and diversity of gut microbes are fundamental for overall body health, in particular for the control of brain physiology [18]. Pathogenic conditions can be induced by a dysbiotic gut microbiota composed of excessive amounts of facultative anaerobes (*Escherichia coli*) and pro-inflammatory *Ruminococcus* or nonbacterial microbes [19].

It is known that gut microbiota–brain host interactions can play a role in mental health, preventing or promoting illnesses such as depression and anxiety [20,21] and acting on mood modulation [22,23]. The complex network of the pathway of the gut-microbiota–brain axis involves the CNS, the autonomic nervous system (ANS), enteric nervous system, gastrointestinal eukaryotic cells, and prokaryotes [24]. Animal studies performed on germ-free mice revealed that with inoculation of the microbiota of patients suffering from major depressive disorders or healthy subjects’ microbiota, they exhibited behaviors related to donors [25]. Comparing depressed individuals with healthy control microbiota, an increased abundance of *Proteobacteria* and *Enterobacteriaceae* that can produce lipopolysaccharides (LPS) has been observed [26], and a positive association among depression, stress response, and *Firmicutes*/*Bacteroidetes* ratio has been advised [27,28]. Unmedicated patients with depression showed microbial signatures such as depletion of *Coprococcus* and *Dialister* and an alteration of the gamma-aminobutyric acid (GABA) biosynthesis.

Recently, Yang et al. [29], through the analysis of 311 human fecal samples, identified 3 bacteriophages, 47 bacterial species, and 50 metabolites with a different abundance between patients with MDD and the healthy control; in particular, the abundance of the genus *Bacteroides* has increased while the abundance of the genera *Blautia* and *Eubacterium* decreased. These changes can be implicated in the pro-inflammatory/anti-inflammatory imbalance in MDD. An alteration of microbial genes and fecal metabolites involved in gamma-aminobutyrate, phenylalanine, and tryptophan metabolism have been identified in MDD patients [29].

Several studies reported neural, endocrine, and inflammatory mechanisms involved in mutual signaling between gut microbiota and the brain [30]. Neurotransmitter metabolism is affected by gut microbiota production of short-chain fatty acids (SCFAs), secondary bile acids, tryptophan metabolites, folate, and GABA [31,32]. Enteroendocrine cells (EECs) and enterochromaffin cells (ECCs) can, in turn, control serotonin release by modifying, through the 5-hydroxytryptamine receptor 2A (HTR2B), liver gluconeogenesis, and glucose uptake [33]. EECs and ECCs release serotonin and other central responses acting via vagal or afferent nerve fibers [34], and several studies demonstrated that the vagus nerve could induce a depression-like phenotype, while subdiaphragmatic vagotomy in antibiotic-treated mice blocked expected changes [35].

Gut microbiota modulate the complex microbiota–gut–brain axis communication by acting via the expression of peptides involved in various biological functions, such as energy metabolism and inflammation [28]. Worthy of particular interest are the molecules that derive from the metabolism of tryptophan, due to their potential neuroactive function [36].

### Tryptophan Metabolism and Gut Microbiota–Brain Axis

Tryptophan is an essential aromatic amino acid introduced with diet, and it is the precursor of serotonin or 5-hydroxytryptamine (5-HT), a monoamine neurotransmitter and consequently of the pineal hormone named melatonin. Serotonin is mainly produced by enterochromaffin cells by the tryptophan hydroxylase 1 (TPH1); an altered expression of this enzyme is reported in psychiatric and gastrointestinal disorders like irritable bowel syndrome (IBS) and inflammatory bowel disorders (IBD), both characterized by psychiatric disorders [37,38]. The gut microbiota is involved with several mechanisms in tryptophan metabolism [39]; some gut bacteria can degrade tryptophan by decarboxylation to tryptamine [40]. Furthermore, tryptophan can also be converted into indole through the enzyme tryptophanase and its derivatives [41].

The commensal gut bacteria promote the colonic serotonin biosynthesis and directly utilize tryptophan to produce serotonin. Tryptophan can be degraded via the kynurenine pathway in the liver, producing kynurenic acid (KYNA) and quinolinic acids (QUIN) with neuroprotective and neurotoxic functions, respectively; these molecules can cross the blood–brain barrier [42]. Therefore, the activation of the kynurenine pathway limits the tryptophan bioavailability for serotonin production and modulates brain functions, influencing neuropsychiatric disorders like depression [43].

Some evidence suggests that microbiota can regulate the expression of kynurenine pathway genes in the hippocampus through micro-RNA-dependent ways, and the SCFAs, such as butyrate, can also modulate kynurenine biosynthesis by the inhibition of the rate-limiting enzyme indoleamine-2,3-dioxygenase [44].

The main metabolites of tryptophan produced by gut microbiota are tryptamine and indolic compounds that can reach distant organs, including the brain. Indole plays a critical role in the gut–brain axis; in fact, an accumulation of this molecule in the brain led to mood disorders and anxiety in animal models [45]. Instead, other microbial indolic derivatives from tryptophan catabolism, including indole, exert an anti-inflammatory action, suppressing CNS inflammation [46].

Some studies have reported the involvement of tryptophan metabolites in major gut disorders, such as IBS, and neuropsychiatric diseases, such as depression and autism spectrum disorders (ASD) [47,48]. In particular, the decreased levels of tryptophan and the increased kynurenine to tryptophan ratio in the plasma are closely related to depression [47], as demonstrated by microbiota transplant experiments from depressive patients to germ-free rats [49].

In conclusion, the metabolism of tryptophan plays an important role in the onset of diseases such as depression and IBS. The gut microbiota, modulating the host tryptophan metabolism, could be an interesting therapeutic approach for the treatment of these pathogenic conditions.

## 3. Oxidative Stress

The term oxidative stress (OS) was originally described as an imbalance between oxidants and antioxidants in favor of oxidants, leading to an interruption of cell signaling, redox control and/or molecular damage [50,51]. OS, in physiological conditions, is connected with inflammation, signaling transduction pathways, immune response, and apoptosis, but a pathological level can be neurotoxic, causing several biomolecular damages due to oxidation of proteins, lipids, and deoxyribonucleic acid (DNA) [50]. The main targets of lipid peroxidation are poly-unsaturated fatty acids (PUFAs) of the membrane, and PUFAs concentration inversely correlates with lifespan in mammals, even if the peroxidation index alone cannot fully explain membrane susceptibility [52].

ROS are mostly free radicals produced by physiological processes occurring in the cell, especially in mitochondria or by interactions with harmful exogenous factors. Depending on their hormetic nature, ROS can trigger different signaling pathways that lead to divergent responses, from adaptation to cell death. Whether a “positive” or “negative” response will prevail depends on many variables, such as the site of ROS production, the persistence of ROS flow, or the antioxidant status of the target cells, among others. Hormesis can be considered an adaptive function characterized by a dose-dependent biphasic response; however, it is tissue-dependent [53]. In particular, CNS requires a high level of ROS due to a high oxidative metabolism and for intra- and intercellular signaling [54]. Free radicals might play a beneficial role in synaptic plasticity, cellular signaling, axon guidance, and apoptosis. Several factors make the brain susceptible to OS; leucocytes and microglia in the brain can also generate ROS, such as H_2_O_2_ or hypochlorous acid, when activated cells increase oxygen consumption during the “respiratory burst”. Most notably, it should be noted that the brain consumes about 20% of the oxygen used by the whole body; furthermore, CNS possesses redox-active transition metals, PUFAs, and auto-oxidating neurotransmitters [54]. In the brain, microglia are considered the primary source of ROS and RNS, as well as TNF-α and glutamate, which are toxic for neuronal cells. In a physiological situation, the effects of ROS production during aerobic metabolism are neutralized by the antioxidant system, and the brain can modulate its oxygen consumption and redox generation capacity. When the scavenging capacity of the antioxidant response system is not able to effectively counteract ROS production, extensive protein oxidation and lipid peroxidation may occur, causing oxidative damage, cellular degeneration, and even functional decline. Oxidative damage in the development of CNS pathologies has been reported for neurodegenerative diseases, including Alzheimer’s and Parkinson’s diseases, amyotrophic lateral sclerosis, demyelinating diseases, depression, and psychiatric disorders [5]. A narrow redox homeostatic window should be maintained for good brain function. The imbalance between an excessive production of ROS and a reduced defense by the endogenous antioxidant systems represents a condition of oxidative stress that triggers, especially at the mitochondrial level. This vicious circle leads the mitochondrion itself to increase ROS production as a result of the damage induced by free radicals, a condition that, in the long term, alters the cellular and physiological balances of the organism [55,56]. This metabolic flexibility, mediated by a protective mechanism in the human being, is also called “redox economy”, which balances the evolutionary tendency of resistance to oxidative stress through the central role of mitochondria in the regulation of adaptive responses [55]. From a cellular biological point of view, this theory is better known as “mitohormesis” [57], according to which oxidative stress depends mostly on ROS produced for the most part by the mitochondrial respiratory chain, capable of favoring adaptive responses rather than representing harmful by-products of oxidative metabolism.

The mitohormetic adaptive response is associated with the responses induced by the gut microbiota: it is in fact recognized an interdependence between gut microbiota and mitochondria by the release of specific metabolites or by endocrine, immunological, and/or humoral signaling molecules [58]. Mitohormesis with ROS production plays a crucial role in regulating the gut microbiota inducing the modulation of the barrier function and both intestinal defense and mucosal immune responses. In this regard, short-chain enteric fatty acids (SCFAs) represent the major metabolites produced by the bacterial fermentation of indigestible carbohydrates and mediate the relationship between the gut microbiota and mitochondria in various organs and tissues. Moreover, branched-chain amino acids (BCAAs), secondary bile acids, nitric oxide (NO), and hydrogen sulfide (H_2_S) are also thought to play at least partial roles in these molecular exchanges between mitochondria and microbiota [59].

Gut microbiota can influence the oxidative state of the CNS, acting on ROS levels and modulating the antioxidant system [60]. Microbiota act on dietary energy harvest, immune system modulation [61], and prevents extensive colonization by pathogenic microbes [62]. They produce vitamins, SCFAs, polyphenols, antioxidant gases, and CNS neurotransmitters (e.g., dopamine, serotonin, and GABA) that can act on CNS, altering the blood–brain barrier (BBB) permeability [63]. Gut microbiota also produce dangerous LPS, amyloid proteins [64], and oxidant gases.

NO is a gaseous molecule that can easily diffuse to tissues, determining pleiotropic effects in the whole organism. It is mainly produced by a family of NO synthetases (NOS) that exist as constitutive isoforms (endothelial cells, platelets, and the nervous system) and inducible ones (macrophages, polymorphonuclear leukocytes, endothelial cells, smooth muscle cells, and hepatocytes). After it has acted, NO is transformed into a series of derivatives, such as nitrites and nitrates, which can be removed from the organism through urine. It can also have dangerous effects when present in excessive amounts and in conditions of increased oxidative stress, since it links to reactive oxygen species to produce highly reactive peroxynitrite (reacting with superoxide [65]), hydrogen peroxide, hypochlorite ions, and hydroxyl radical. In physiological amounts, NO regulates important functions in the vascular endothelium, immune system, and gastrointestinal motility and, in the brain, it is involved in the control of learning and memory, guarantying synaptic function, blood supply, and regulating neuronal function by glycosylation. Furthermore, in environmental conditions favoring peroxynitrite formation, neuroinflammation and neurodegenerative disorders may occur. In the gut, NO is produced by intestinal tissues and leukocytes but also by microbial bacteria that can synthesize NO starting from nitrate and nitrite, linking nitrate/nitrite/NO metabolism to diet and health through microbiota [66]. Gut microbiota dysbiosis has been associated with inflammatory disease, which causes elevated NO levels, and is dangerous for brain health. Leclerc et al. [67] demonstrated that excessive NO impacts human gut microbiota diversity, favoring species that can sustain high NO concentrations instead of healthy ones, supporting a vicious circle of inflammation.

An inter-talk between host and microbiota mediated by mitochondria [68], representing the primary ROS and RONS production site, has been demonstrated. Gut microbiota can reduce mitochondrial ROS production and its effects [58] via SCFAs. On the contrary, *Salmonella* and *E. coli* can produce H_2_S in the gut, a metabolite that inhibits COX activity and shifts the metabolism to lactate production [69], reduces mitochondrial activity, and induces inflammatory molecules, such as IL-6.

Another highly diffusible molecule is molecular hydrogen (H_2_, dihydrogen), which possesses neuroprotective effects; it is generated by microbiota during fermentation, despite not being produced directly by humans [70]. Butyrate, principally derived from the enteric microbiome, is known to modulate mitochondrial function, and has been proposed as a neuroprotectant in pathologies involving mitochondrial dysfunction, such as autism spectrum disorders [71]. Furthermore, microbiota-produced LPS can reach the CNS via the vagus nerve (VN) or systemic circulation, leading to microglial activation, neuroinflammation, and ROS production [54]. Healthy gut microbiota have an essential antioxidative and anti-inflammatory role in protecting the CNS from neurodegeneration mediated by oxidative stress.

In physiological conditions, gut epithelium produces free radicals that can modify gut microbiota composition and functionality and intestinal permeability, allowing xenobiotic molecules to reach the systemic circulation and the CNS [72]. On the other hand, CNS can act on the intestine via the vagus nerve [73], suggesting that dysbiosis may induce ROS generation in the brain, and can, in turn, be a result of it.

## 4. Dysbiosis and Depression

The neurobiological changes linked to depression include alterations of neurotransmitter levels and the hypothalamic–pituitary–adrenal (HPA) axis, leading to inflammation [74]. As previously described, the inflammatory pathway associated with depression is characterized by an increase in ROS and RNS, with a reduction in endogenous antioxidants and neurodegeneration, and decreased neurogenesis and neuroplasticity [75]. Gut microbiota can play an essential role in oxidant–antioxidant balance: in fact, it has been demonstrated that germ-free mice present reduced antioxidant enzyme activity [76].

Gut dysbiosis, with a prevalence of bacteria associated with depression, can induce depressed mood-producing isovaleric acid [77], which possesses a direct effect, or promoting the kynurenine production, as previously described, thus acting in an indirect manner [49]. Irritable bowel syndrome is a common gastrointestinal disorder causing abdominal pain and irregular defecation, associated with changes in gut microbiota. In order to understand IBS depression comorbidity, Han et al. [78] analyzed serum/fecal metabolome alterations related to microbiota in IBS patients. A quantity of 726 differentially abundant serum metabolites, including a cluster of fatty acyl-CoAs enriched in IBS, have been identified. Furthermore, three species, including *Odoribacter splanchnicus*, *E. coli*, and *Ruminococcus gnavus*, were strongly associated with the low abundance of dihydropteroic acid. A dysregulated tryptophan/serotonin metabolism correlated with the severity of IBS depression in both fecal and serum metabolomes, characterized by a shift in tryptophan metabolism towards kynurenine production.

Lai et al. [79] reported that depressed patients with bipolar disorder following quetiapine monotherapy have specific alterations in gut microbial diversity and composition. The abundance of *Clostridium bartlettii* was negatively associated with age and baseline depression severity, while positively associated with spontaneous neural oscillation in the hippocampus.

Even if antibiotic exposure leads to dysbiosis, potentially increasing the risk of depression, short-term ciprofloxacin and metronidazole administration can reverse chronic unpredictable mild stress-induced depression-like behavior [80].

In pathological conditions, gut microbiota can cross the epithelial barrier, reaching the mesenteric lymph nodes and triggering an immune response that culminates in the production of lysozyme [81] and, therefore, in the destruction of bacteria.

The heat shock proteins (HSP) belong to a class of intracellular proteins that, ubiquitously expressed in physical and psychologically stressful conditions, repair and stabilize proteins, protecting the gut epithelial barrier from OS and inflammation. Furthermore, the excessive production of HSP, or their liberation after apoptosis or cell death, can stimulate a pro-inflammatory response [82]. In a longitudinal study on neurotropic, oxidative, and inflammatory markers in depressed midlife women, elevated levels of HSP70 have been reported [83], and gut microbiota have been demonstrated to modulate HSP, since *Lactobacilli* induce gut protection through gut epithelial HSP modulation [84]. Gut microbiota can modify levels of adrenocorticotropic hormone (ACHT) [85] and corticosterone [86], which are depression-sustaining substances. Human studies in depressed patients reported higher *Bacteroidetes* and lower *Lachnospiraceae* proportions, alongside reduced microbial diversity [22]; furthermore, chronically depressed patients present high serum levels of immunoglobulin (Ig) A and IgM antibodies against the LPS of *Enterobacteriaceae*, sustaining a chronic inflammatory status [87]. Due to the potential of probiotics to decrease the plasmatic cortisol level, with the reduction of depressive symptoms, the use of specific treatments aimed to improve gut microbiota health in preventing and counteracting depression should be considered.

## 5. Antidepressant Effects of Nutraceuticals

The richness and diversity of the gut microorganisms are important for general host health and the prevention of mental diseases, such as depression and anxiety. Some foods and molecules can affect gut microbiota, leading to a healthy microbial composition [88].

Nutraceutical supplements, such as probiotics, polyphenols, carotenoids, butyrate, and prebiotics, that seem to exert an antidepressant activity, and the possible communication pattern between brain and gut microbiota, will be discussed.

### 5.1. Psychobiotics

Psychobiotics are defined as live organisms that, when ingested in adequate amounts, confer mental health benefits to the host through interaction with commensal gut bacteria [89]. Even if the mechanism of action of these bacteria has not been completely elucidated, it is probable that it may be mediated through the hypothalamic–pituitary–adrenal (HPA) axis, the immune response and inflammation, and through the production of neurohormones and neurotransmitters.

Several neurotransmitters and proteins, including GABA, serotonin, glutamate, and brain-derived neurotrophic factor (BDNF), are involved in controlling neural excitatory-inhibitory balance, mood, cognitive functions, learning, and memory processes and might be regulated by psychobiotics. Up to now, the majority of psychobiotics research has been performed using animal models [90].

Sudo and colleagues [91] investigated the HPA response to stress by comparing genetically identical mice that had no exposure to microorganisms (germ-free; GF), mice raised with normal functional microbiota but not with specific pathogens (specific pathogen-free; SPF), and mice raised with a selected group of organisms (gnotobiotic): results showed that commensal microbiota could affect the postnatal development of the HPA stress response in mice.

Several probiotic strains were reported as psychobiotics from animal studies, having psychotropic effects on depression, anxiety, and stress, due to their ability in producing and delivering neuroactive substances, such as GABA and serotonin, which act on the brain–gut axis.

Serotonin is a key neurotransmitter in the brain–gut axis influencing behavior, and approximately 95% of serotonin is derived from enterochromaffin gut cells and ENS neurons, which are associated with the regulation of GI secretion and motility. The development of the gut microbiome overlaps with the ontogeny of the serotonergic system; for this reason, the gut microbiota are an appealing therapeutic target for gut–brain axis disorder.

*Lactobacillus brevis*, *Bifidobacterium dentium*, and *Lactobacillus plantarum* produce GABA and serotonin [31], as well as *L. plantarum* and *Lactobacillus odontolyticus*, which can produce acetylcholine [92].

Recently, Yano et al. [93] have demonstrated that microbes can regulate serotonin synthesis in the gut. For instance, spore-forming bacteria from the gut microbiota have been found to induce serotonin biosynthesis from gut enterochromaffin cells. With the knowledge acquired in this field, Dinan et al. [89] proposed that the application of psychobiotics may require a precision strategy for targeting anxiety and depression behaviors. Liu et al. investigated *L. plantarum* PS128 and its role in reducing the anxiety and depression-like behaviors of mice. PS128 reduced inflammatory cytokine levels and increased anti-inflammatory cytokine levels in the serum of mice, significantly decreasing inflammation and corticosterone levels. Notably, administration of PS128 significantly increased dopamine and serotonin levels in the prefrontal cortex and striatum compared with control mice [94]. Likewise, animal studies on rats have shown that the administration of the single strain *Lactobacillus helveticus* NS8 reduced anxiety, depression, and cognitive dysfunction. In addition, it increased the serotonin, norepinephrine (NE), and BDNF levels in the hippocampus [95]; additionally, a single strain of *Bifidobacterium longum* 1714 decreased stress, depression, and anxiety behaviors [96]. The chronic treatment with *Lactobacillus rhamnosus* JB-1 in mice led to an increase of GABAB1b expression in cortical regions (cingulate and prelimbic) of the brain, and a decreased expression in the hippocampus, amygdala, and locus coeruleus, in comparison with control-fed mice [97]. GABAAα2 mRNA expression has also decreased in the prefrontal cortex and amygdala, and increased in the hippocampus. GABA is the main inhibitory neurotransmitter of the CNS, and central GABA receptor expression alterations are implicated in the pathogenesis of anxiety and depression. For this reason, these receptors are important pharmacological targets for clinically relevant antianxiety agents (e.g., benzodiazepines acting on GABA receptors), and alterations in the GABAergic system have important roles in the development of stress-related psychiatric conditions. Furthermore, Bravo et al. [97] identified the vagus as one of the principal modulators between the bacteria exposed to the gut and the brain. JB-1 treatment did not reduce stress-induced corticosterone, anxiety- and depression-related behavior in vagotomized mice [97].

The administration of the single strain *B. longum* NCC3001 effectively treated anxiety, upregulating the expression of BDNF in the hippocampus [98].

A treatment with *Bacterium infantis* 35624 resulted in the normalization of the immune response, reversing behavioral deficits, and restoring basal NE concentrations in the brainstem [99]. *B. infantis* 35624 was found to be effective on depression-like behaviors.

*Lactobacillus johnsonii* BS15 could prevent memory dysfunction in mice induced by psychological stress through modulating the gut environment, including intestinal inflammation and permeability. In the intestines, *L. johnsonii* BS15 enhanced the mRNA levels of tight junction proteins and exerted beneficial effects on the anti-inflammatory cytokine levels [100]. In addition to promising animal studies, several researchers have found the positive effects of probiotics on mental health in humans.

One of these studies examined healthy volunteers who were given a *B. longum* 1714 for four weeks, and the result exhibited reduced stress and improved memory [101].

The benefits of probiotic yogurt (*Lactobacillus acidophilus* LA5 and *Bifidobacterium lactis* BB12) and probiotic capsules (*Lactobacillus casei*, *Lactobacillus acidophilus*, *Lactobacillus rhamnosus*, *Lactobacillus bulgaricus*, *Bifidobacterium breve*, *Bifidobacterium longumand*, and *Streptococcus thermophilus*) supplementation on petrochemical workers were studied for six weeks in a randomized, double-blind, placebo-controlled experiment [102]. Recipients using probiotic yogurt and probiotic capsules exhibited improved mental health parameters, measured using the Depression, Anxiety, and Stress Scale (DASS) and the General Health Questionnaire (GHQ). The consumption of probiotic yogurt or a multispecies probiotic capsule had beneficial effects on mental health parameters in petrochemical workers. A probiotic combination of *L. helveticus* R0052 plus B. longum R0175 reduced anxiety and depression in healthy subjects compared with the control ones [103]. Even if there is promising evidence on the positive effects of psychobiotics in human studies, further clinical studies are needed [104,105].

### 5.2. Prebiotics

Nurturing a beneficial gut microbiome with prebiotics, such as fructo-oligosaccharides (FOS) and galacto-oligosaccharides (GOS), is an appealing but under-investigated microbiota manipulation. These prebiotics are the preferred sources of nutrition for *Bifidobacteria* and *Lactobacilli*, which probably mediate their neurological and cognitive effects.

Burokas et al. tested whether chronic prebiotic treatment modifies behavior across domains relevant to anxiety, depression, cognition, stress response, and social behavior [106]. Chronic prebiotic FOS+GOS treatment exhibited both antidepressant and anxiolytic effects in mice. Moreover, the administration of GOS and the FOS+GOS combination reduced stress-induced corticosterone release. Prebiotics modified specific gene expression in the hippocampus and hypothalamus. Regarding SCFA concentrations, prebiotic administration increased cecal acetate and propionate and reduced isobutyrate concentrations, which significantly correlated with the positive effects on behavior. Moreover, FOS+GOS reduced chronic stress-induced elevations in corticosterone and pro-inflammatory cytokine levels and depression-like and anxiety-like behavior in addition to normalizing the effects of stress on the microbiota [106].

### 5.3. Polyphenols

Polyphenols, one of the essential micronutrients in the human diet, protect human health and have recently gotten a lot of attention. The antidepressant effect of polyphenols occurs through different mechanisms due to the variety of sources and types of polyphenols, and involves more than just their well-known antioxidant properties. The main mechanisms of polyphenols reducing depression are related to lowering oxidative stress and neuroinflammation, and increasing neurotrophin release. The processes proposed seem to also have an impact on serotonin, norepinephrine, and/or dopaminergic systems regulation to influence the HPA axis and modify the concentration of the BDNF [107], mitogen-activated protein kinase phosphatase-1 (MKP-1), and cAMP-responsive element-binding protein (CREB), and to normalize phosphorylation levels in the hippocampus, prefrontal cortex-modulating synaptic plasticity, activation of neuroplasticity, neurogenesis, and cell survival signaling pathways (PKA, CaMKII, PKC, MAPK/ERK, and PI3K) [108]. The use of dietary polyphenols to target various cell signaling pathways related to oxidative stress and inflammation, including MAPK, NFκB, and PI3K/Akt, is becoming a new method for preventing and treating depressive disorders. Polyphenols regulate the MAPK signaling pathway by acting on various steps in the activation process. This evidence suggests that the influence of polyphenols on depression-related psychiatric diseases needs to be further investigated.

Polyphenols in the colon cause a series of modifications in gut microbiota, influencing absorption and bioavailability. These molecules promote the proliferation of beneficial, and inhibit that of harmful, bacteria, regulating gut microbiota composition and thus maintaining gastrointestinal health. Meanwhile, the gut microbiota have a fundamental role in the health-promoting effects of polyphenols.

*Bifidobacteria*, *Lactobacilli*, *Bacterobacter*, *Bacillus*, *E. coli*, and others are substrates for dehydrogenation, esterification, glycosylation, demethylation, and decarboxylation reactions performed by polyphenols. The latter are thus beneficial to produce smaller colon products, such as phenolic acids and other polyphenol-related bacterial metabolites.

It is widely recognized that the host’s metabolism, immunological function, and food absorption are all influenced by the gut microbiota, and the disruption of balanced gut microbiota can have a significant negative influence on health [109].

Polyphenols—or foods rich in polyphenols—can alter the gut microbiota composition due to their “prebiotic-like” action [110,111], and their regulatory role is primarily based on two factors: first off, polyphenols themselves have antibacterial properties; secondly, polyphenols augment the flora-dependent nutritional supply. For example, in animal models, modifications of intestinal microflora structure and energy conversion genes have been reported after the administration of green tea polyphenols; these polyphenols improved vitamin production and affected amino acid metabolism patterns, reducing caloric carbohydrates, cholesterol, and cholic acid in intestinal microbial metabolites [112]. Furthermore, several compounds contained in tea are effective modulators of dopaminergic activity. Overall, numerous molecules present in all major tea types (predominantly l-theanine, and polyphenols and associated metabolites) are capable of lowering the risk of depression, by simultaneously functioning through multiple pathways collectively [113].

The microbiota–gut–brain axis is a comprehensive physiological system responsible for the connection between the gastrointestinal system and the brain. Its dysfunction or imbalance, associated with various immune, neurological, and psychiatric diseases, has been previously discussed extensively [114]. Therefore, the influence of polyphenols on the microbiota–gut–brain axis, and particularly on the HPA axis, might be one of the ways to regulate depression.

Polyphenols appear to be an effective neuroprotective agent, according to emerging data. In Wistar rats, quercetin and diets high in polyphenols reduced the mRNA expression of corticotropin-releasing factor (CRF) in the hypothalamus area, inhibiting the HPA axis activation generated by acute water immersion restraint, and hence resulting in decreased depressive-like behavior [115].

Resveratrol, another polyphenolic molecule with various functions, has received much interest because of its potential usefulness in preventing and treating depression through regulating the HPA axis activity in the peripheral nervous system [116]. In addition, some evidence suggests that the HPA axis activity may also be related to the antidepressant effect of curcumin in chronically stressed animals.

Further, the well-functioning of the immune system is strongly related to human health; its dysfunction is a major cause of many diseases, including MDD [117]. The immune system regulates the gut bacteria population while being influenced by the gut environment and the CNS. The main mechanisms by which polyphenols regulate the immune system are the activation of immune cells and their influence on epigenetic mechanisms. As a matter of fact, many studies show that polyphenols from various sources can modulate the immune system in distinct immune cell types. In mice given polyphenol-rich extracts from the date palm tree, significant increases of immunocompetent cells in the spleen and Peyer’s patch were found [118]. A similar effect has been observed with cocoa, which is able to regulate the immune response in rats by reducing IgA secretion in the intestine [119]. On the other hand, microRNA-driven epigenetic alterations are all implicated in immune regulation and affect the gene expression of important molecules related to the immune response [120]. Polyphenols can also influence epigenetic patterns by modifying the levels of *S*-adenosylmethionine and *S*-adenosylhomocysteine, as well as the enzymes that catalyze DNA methylation and histone modifications. The epigenome is influenced by the polyphenol EGCG, which inhibits DNA methyltransferase-1 (DNMT1) and gene transcription [121].

Researchers proposed the “microbiota–gut–inflammasome–brain” hypothesis of MDD, according to which intestinal microbial dysbiosis can cause an upregulation of the pro-inflammatory pathway mediated by the NLRP3 inflammasome, worsening depression symptoms while further aggravating gut dysbiosis [122].

The gut microbiome is also involved in the production of melatonin, GABA, catecholamine, acetylcholine, histamine, and 5-HT. The latter is not only involved in emotion regulation but also in secretion, perception, and signal transduction in the gastrointestinal tract. As a result, 5-HT is an important signaling molecule in the microbiota–gut–brain axis. In enterochromaffin epithelial cells (ECs), it is mainly produced by tryptophan; polyphenols alter the activity of indoleamine-2,3-dioxygenase, directly affecting tryptophan metabolism and thus 5-HT production [123].

Moreover, the gut microbiota play a key role in regulating the bioavailability level of tryptophan and the subsequent synthesis of 5-HT. Modifications in 5-HT levels appear to be mediated by the release of small molecules (e.g., SCFAs), which signal ECs to produce 5-HT via tryptophan hydroxylase expression. As a result, polyphenols indirectly increase 5-HT levels by increasing the intestinal microbiota’s production of SCFAs. In addition, depression is linked to an imbalance in GABA signaling. *Bifidobacterium* produces GABA through the enzymatic decarboxylation of glutamate in rats. *L. rhamnosus* JB-1 was also found to reduce the anxiety and depression-like behavior in mice in a vagus nerve-dependent manner, accompanied by changes in the brain GABA activities. In humans, preliminary reports have also proposed that human intestinal microbiota, especially some strains of *Lactobacillus* and *Bifidobacterium* (*Lactobacillus brevis* and *Bifidobacterium dentium*), can generate GABA by metabolizing glutamate in the diet [124].

Another key mechanism linking polyphenols to depression is the effect of bacterial metabolites related to polyphenols. As an example, the main metabolites of chlorogenic acid are ferulic acid, m-cumaric acid, phenyl propionic acid, benzoic acid, and hippuric acid derivatives [125]. Ferulic acid was reported to exert antidepressant effects in animal models by improving the monoaminergic system, the antioxidant defense mechanism, the regulation of inflammatory and apoptotic signaling pathways, and the healing of stress damage induced by HPA axis malfunction [126,127].

Caffeic acid, rosmarinic acid’s main metabolite, was shown to reduce immobility time in the forced swimming test without affecting monoamine uptake or oxidase activity, thus suggesting that caffeic acid might have a different antidepressant mechanism than the ones currently used in the clinic [128].

Furthermore, human gut microbiota transform ellagic acid into urolithins, a group of small molecular compounds produced by the *Gordonibacter* species, which have a variety of biological actions, the most well-known of which is anti-inflammatory activity. Urolithins are more easily absorbed than their predecessors and were found to pass the blood–brain barrier, and have a beneficial effect on neurological illnesses [129].

Polyphenols, through their probiotic effects on the gut microbiota, such as *Bacteroidetes* and *Firmicutes*, induce the formation of SCFAs. Chlorogenic acid, caffeic acid, rutin, and quercetin have all been shown to promote the formation of SCFAs, such as propionate, butyrate, and acetate [130]. However, more animal and human clinical trials are needed to study the antidepressant effects of gut microbiota and polyphenols.

### 5.4. Short-Chain Fatty Acids

SCFAs are small organic molecules produced by the anaerobic fermentation of mostly indigestible dietary carbohydrates in the cecum and colon, which cross-feed other bacteria and are easily absorbed in the large bowel. SCFAs are involved in digestive, immune, and central functions, although different accounts on their impact on behavior exist.

The three most common SCFAs (acetate, butyrate, and propionate) have been shown to alleviate depressive symptoms in mice, and the antidepressant properties of these molecules have also been discussed in studies by Caspani et al. [131] and Silva et al. [132], research which suggested a crucial role in neuro-immunoendocrine regulation even if the mechanisms have not been fully elucidated. A decrease of butyrate, acetate, and propionate has been documented in MDD patients, indicating their role in the genesis of depression [133]. In addition, patients with greater quality of life markers had a larger abundance of butyrate-producing bacteria, such as *Faecalibacterium* and *Coprococcus* spp.

SCFAs play a role in the production and release of peripheral neurotransmitters (such as 5-HT and acetylcholine) by enterochromaffin cells, as well as norepinephrine by sympathetic neurons, as previously indicated. The SCFA propionate also works as an HDAC inhibitor, and sodium propionate was given intrarectally to rats to ameliorate their despair behavior. The propionate antidepressant effect was accompanied by increases in norepinephrine, dopamine, tryptophan, 5-HIAA, and 3-hydroxyanthranilic acid (3-HAA) in the prefrontal cortex, but no change in 5-HT was seen [134,135]. Propionate dysregulation has been widely documented in animal models of depression, but its neurotoxic effects and behavioral abnormalities produced at high dosages suggest that greater information of the underlying pathways is needed before a focused remedy can be created [134].

Acetic acid has also been demonstrated to impact the availability of histone acetyltransferase substrates, which are important for epigenetic control. Furthermore, butyrate affects multiple host physiological processes via specific transporters/receptors, and as a histone deacetylases (HDACs) inhibitor it causes histone acetylation and stimulates gene expression in host cells. Histone acetyltransferases (HATs) and histone deacetylases (HDACs) are catalytic enzymes that are attractive targets for the therapy of neurodegenerative disorders and cognitive decline [136]. For these reasons, butyrate has been used as an experimental drug in models for neurological disorders, suggesting that butyrate’s physiological levels may indirectly influence the brain via regulating the immune system and vagus nerve activity. Supraphysiological doses of butyrate exert potent neuropharmacological effects, facilitating synaptic tagging and capturing [137].

More importantly, the direct influence of SCFAs on gastrointestinal cells leads to the synthesis of hormones like leptin, and stimulates leptin secretion, supporting the central nervous system and indirectly playing an antidepressant role.

### 5.5. Omega-3 Poly-Unsaturated Fatty Acids (PUFAs)

A growing body of evidence has indicated that omega-3 poly-unsaturated fatty acids (omega-3 PUFAs) have been effective in improving depression [138,139]. Supplementation with the two main types of omega-3 PUFAs, eicosapentaenoic acid (EPA) [140] and docosahexaenoic acid (DHA) [141], has also been found to be effective in reducing the symptoms of depression. Omega-3 poly-unsaturated fatty acids (PUFAs) have been proposed as a treatment for MDD. Over the last decade, several meta-analyses have been performed, which suggested variable degrees of the beneficial effects of omega-3 PUFAs for MDD, but which made critical remarks regarding the quality of the evidence and possible publication bias. Mocking et al. [139] evoked academic correspondence, discussing the used inclusion criteria and the selection of outcome measures.

In brief, this correspondence suggested beneficial effects. Firstly, if a higher ratio of omega-3 PUFA eicosapentaenoic acid (EPA) to docosahexaenoic acid (DHA) was being supplemented, and secondly, only in patients with actual MDD as opposed to subjects with merely depressive symptoms [139].

### 5.6. Vitamin A and Carotenoids

Vitamin A (VA) and its derivatives have an important role in the development of the CNS [142] and are fundamental for normal learning and memory functions [143]. VA and β-carotene levels in patients with Alzheimer’s disease (AD) are significantly lower than those in normal controls, and Vitamin A deficiency (VAD) aggravates cognitive impairment and plays an essential role in the pathogenesis of AD. In order to clarify the role of gut microbiota in VAD cognitive function, Chen et al. [144] analyzed the effect of a VA-deficient diet for 45 days in twenty 8-week-old male C57BL/6J amyloid precursor protein/presenilin 1 (APP/PS1) transgenic mice, showing that VAD aggravated the behavioral learning and memory deficits, and decreased the expression of GABA receptors and the liver and serum retinol. VAD increased the morphological, histopathological, molecular biological, microbiological, and behavioral impairment in the APP/PS1 transgenic mice and altered the gut microbiota’s composition and functionality, suggesting that VA has a key mediator role in this mechanism [144]. Furthermore, dietary carotenoids, such as β-carotene and astaxanthin, have been shown to improve immunoglobulin A expression, and the gut immune system maturation, with the consequent promotion of gut health [145].

The natural carotenoid crocin, contained in saffron and gardenia flowers (crocuses and gardenias), exhibits a variety of pharmacological effects, including an anti-inflammatory action due to the reduction of lipopolysaccharide (LPS), Interleukin-6, and tumor necrosis factor-α (TNF-α) levels in serum, and TNF-α expression in the hippocampus [146], as well as a neuroprotective function [147], increasing the hippocampal brain-derived neurotrophic factor, with possible potential to treat depression [148,149].

Crocin-I supplementation (40 mg/kg for six weeks) decreased the gut microbiota dysbiosis in depressed mice, represented by the decreased abundance of *Proteobacteria* and *Bacteroidetes*, *Sutterella* spp. and *Ruminococcus* spp., and increased abundances of *Firmicutes*, *Lactobacillus* spp., and *Bacteroides* spp., also with an increase of SCFAs. These results suggested that crocin-I effectively alleviated depression-like behavior, likely depending on the gut microbiota and its modulation of the intestinal barrier and SCFAs [150].

A preventive effect of lycopene (LYC), a functional carotenoid component, on colitis and the accompanying behavior disorders has been reported [151]. In this study, the LYC treatment (50 mg/kg body weight/day) for 40 days prevented dextran sulfate sodium (DSS)-induced gut barrier damages and inflammatory responses in male mice. LYC improved DSS-induced depression and anxiety-like behavioral disorders by suppressing neuroinflammation and preventing synaptic ultrastructure damages by upregulating the expressions of neurotrophic factor and postsynaptic-density protein. Moreover, LYC reshaped the gut microbiome in colitis mice by decreasing the relative abundance of *Proteobacteria* and increasing the relative abundance of *Bifidobacterium* and *Lactobacillus*. LYC also elevated the production of SCFAs and inhibited the permeability of lipopolysaccharide in colitis mice. In conclusion, LYC ameliorates DSS-induced colitis and behavioral disorders via mediating the microbes−gut−brain axis balance [151]. An inverse association between total carotenoid (alpha-carotene, beta-carotene, beta-cryptoxanthin, lycopene, and lutein with zeaxanthin) intake with the risk of depressive symptoms in U.S. adults has also been suggested by Ge et al. [152]. A summary of the studies which investigated associations between nutraceuticals and MDD is presented in Table 1.

## 6. Exercise as an Antidepressant through the Microbiota–Gut–Brain Axis

Regular exercise has been previously shown to be particularly beneficial in reducing stress levels and has also been proposed as an effective antidepressant method, although its mechanisms are still unclear [155]. Some consistent evidence suggests that exercise might alleviate depression by acting on neuromolecular mechanisms, such as increased serotonin availability, HPA-axis activity regulation, and expression of neurotrophic factors [156].

**Figure 1:**
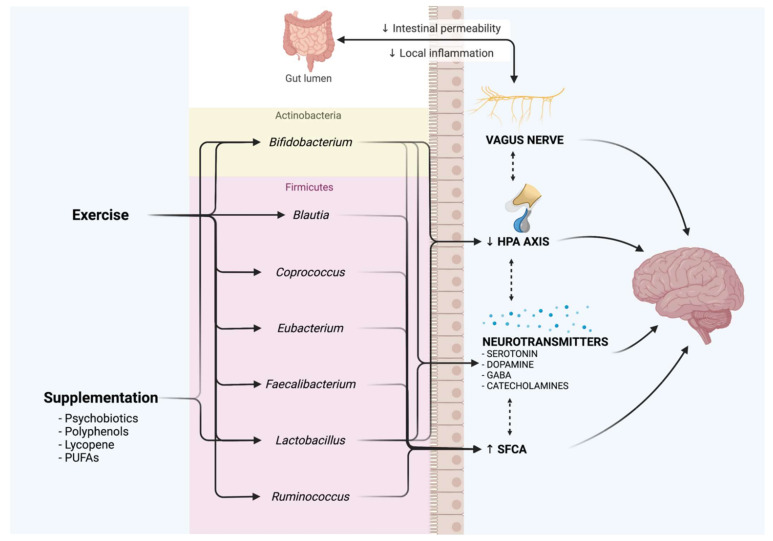
Effect of exercise and supplementation on the main microbiota genera associated with depressive disorders. Both exercise and supplementation cause an increase in the abundance of all the genera presented, which play a role in the communication between the gut and nervous system through the microbiota–gut–brain axis. Main pathways of action are also presented. Created with BioRender.com.

It was recently established that the gut and the brain can communicate through these pathways, with gut microbiota influencing this connection [157]; thus, it has been hypothesized that physical exercise might alleviate stress-related and depression symptoms through a modification of the gut microbiota composition and function, and this thesis is confirmed by some studies conducted on rats [158]. Indeed, data suggest that gut microbiota could strongly contribute to the benefits that exercise exert on brain function, although the underlying mechanisms remain unclear [159]. In humans, numerous studies showed a positive correlation between individuals’ levels of fitness and microbiome diversity, and interestingly, improvements in physical exercise and mental health are associated with the alterations of some taxa in the gut [159,160,161]. Exercise promotes the increase in microbiota α diversity and richness, and the abundance of beneficial bacteria species. Donati Zeppa et al. [162] showed a significant increase in the *Firmicutes*: *Bacteroidetes* ratio and the abundance of *Actinobacteria* phylum, while observing a reduction of *Proteobacteria* phylum. At the genus level, several taxa that have been previously reported as being associated with depressive-like behaviors also showed an association with increased cardiovascular fitness or in response to a training period (i.e., reduced abundances in case of depression, increased abundances in case of higher fitness level). In particular: increased levels of *Bifidobacterium*, *Blautia*, and *Lactobacillus* were reported in rats with free access to exercise with respect to a non-exercise group [163]; Donati Zeppa et al. [162] showed an increase in *Blautia*, *Bifidobacterium*, and *Ruminococcus* in response to 9 weeks of high-intensity training in healthy young males; increased abundance of *Faecalibacterium* and *Roseburia* have been reported by Allen et al. [164] after 6 weeks of endurance training in sedentary subjects; abundance of *Coprococcus* was associated with exercise frequency by McFadzean R. [165]; and *Prevotella* and *Methanobrevibacter smithii* were reported to be increased in a sample of competitive cyclists, with *Eubacterium* genus being more abundant in a specific sub-group of cyclists characterized by higher richness and alpha diversity [166]. These modifications have an impact on the microbiota–gut–brain axis through different mechanisms, such as activation of the vagus nerve, the modulation of neurotransmitters metabolism (i.e., tryptophan, which is converted and produces over 90% of the serotonin in the gut), the regulation of the HPA-axis, an increase of SCFAs production (and thus inflammation reduction), and gut hormones (i.e., GABA, neuropeptide Y, and dopamine, that act locally on the enteric nervous system) [159,167]. Indeed, some evidence suggests that a possible mechanism through which exercise might be beneficial in the control and treatment of depression is the ability of the gut microbiota to regulate tryptophan metabolism (via the kynurenine pathway), which is strongly associated with depression [14,168]. An overview of the exercise-induced microbiota modifications at the genus level and the possible mechanisms of action in managing depressive symptoms are presented in Figure 1.

The microbiota–gut–brain axis is bidirectional: the modifications in microbiota composition affect behaviors (e.g., depression), and interventions that affect behavior (e.g., exercise) through several mechanisms result in changes in the microbiota. However, the extent to which exercise effects the gut–brain axis mediated by alterations in the microbiome remains unknown. Moreover, exercise-induced modifications on the gut microbiota, and thus the effects on the gut–brain axis pathways, are specific to the duration and intensity of the exercise performed; consequently, further research is needed to assess which exercise model could be preferred in terms of antidepressant effects.

## 7. Conclusions

The potential mechanisms proposed at the base of the positive association between gut microbiota health and mental well-being are strictly connected. There are no unique dietary elements or nutritional approaches for improving mental health conditions, even if a general group of nutraceuticals seems to have potential beneficial effects in preventing mental health disorders through synergistic interactions with the gut microbiota. The major nodes within the network framework of depression include HPA axis hypersensitivity, inflammation, debilitated monoaminergic systems, decreased neurogenesis/neuroplasticity, and a limited microbiome diversity.

Such emerging evidence emphasizes the critical role of microbiota in the gut–brain cross-talk and suggests that certain bacterial clusters can be useful therapeutic coadjutants in stress-related CNS disorders, with particular attention paid to the role of some of the major metabolites produced by bacteria, such as SCFAs and other nutraceuticals, in regulating neuro-immunoendocrine function.

## Figures and Tables

**Table 1:** Summary of studies on putative antidepressant nutraceuticals, gut microbiota and MDD.

Category	Treatment	Possible Mechanism of Action	Model	Reference
Probiotics	*Lactobacillus brevis*	↑ GABA	Human	[31]
	*Bifidobacterium dentium*	↑ GABA ↑ 5-HT	Mice	[31]
	*Lactobacillus plantarum*	↑ GABA ↑ 5-HT ↑ ACh	Mice	[31]
	*Lactobacillus odontolyticus*	↑ ACh	Mice	[92]
	*Lactobacillus plantarum* PS128	↓ Pro-inflammatory cytokine ↑ Anti-inflammatory cytokine ↑ DA ↑ 5-HT	Mice	[94]
	*Lactobacillus helveticus* NS8	↑ BDNF ↑ NE ↑ 5-HT	Sprague–Dawley rats	[95]
	*Lactobacillus rhamnosus* (JB-1)	↑ GABA B 1b (cortical regions) ↓ GABA B 1b (hippocampus, amygdala, and locus coeruleus) ↓ GABA A α2 (prefrontal cortex and amygdala) ↑ GABA A α2 (hippocampus)	Adult male BALB/c mice	[97]
	*Lactobacillus johnsonii* BS15	↑ Anti-inflammatory cytokine	C57BL/6J male mice	[100]
	*Bifidobacterium longum* 1714	-	Healthy human males	[101]
	Probiotic yogurt (*Lactobacillus acidophilus* LA5 and *Bifidobacterium lactis* BB12)	↑ Mental health (DASS, GHQ)	Human	[102]
	Probiotic capsule (*Lactobacillus casei*, *L. acidophilus*, and *L. rhamnosus*)	↑ Mental health (DASS, GHQ)	Human	[102]
Prebiotics	Fructo-oligosaccharides (FOS) Galacto-oligosaccharides (GOS)	↑ Acetate ↑ Propionate ↓ Isobutyrate ↓ Pro-inflammatory cytokine	C57BL/6J male mice	[106]
PUFAs	Eicosapentaenoic acid (EPA) Docosahexaenoic acid (DHA)	-	Human	[139]
SCFAs	Propionate	↑ NE ↑ DA ↑ TPH	Sprague–Dawley rats	[134,135]
Polyphenols	Quercetin	↓ CRF ↓ HPA activation ↑ SCFAs	Wistar rats	[115]
	Resveratrol	Neuroprotective	Sprague–Dawley rats	[116]
	Ferulic acid	↓ MAO-A ↑ Antioxidant defense ↑ Monoaminergic system ↑ SCFAs	Mice	[126,127]
	Caffeic acid	↓ MAO ↓ Monoammine uptake ↑ SCFAs	ICR Mice	[128]
	Rosmarinic acid	↓ Corticosterone ↑ Dopamine ↓ Mkp-1 ↑ BDNF	Mice	[153]
	Ellagic acid	↑ 5-HT ↑ NE	Mice	[154]
	Quercetin, Chlorogenic acid, and Caffeic acid	*Bifidobacterium* *Bacteroidetes* *Firmicutes*	Human	[130]
Tea	Tea saponin, l-theanine, Epigallocatechin gallate, and Catechine	↓ NF-kB ↑ DA	-	[113]
Lycopene	-	↓ Neuroinflammation ↑ Neurotrophic factor ↑ SCFAs ↑ *Bifidobacterium* ↑ *Lactobacillus*	Mice	[151]

Note: 5-HT: serotonin; ACh: acetylcholine; BDNF: brain-derived neurotrophic factor; CRF: corticotropin-releasing factor; DA: dopamine; DASS: depression anxiety stress scales; GABA: gamma-aminobutyric acid; GHQ: Global Health Questionnaire; HPA: hypothalamic–pituitary–adrenal axis; MAO: monoamine oxidases; Mkp-1: mitogen-activated protein kinase phosphatase-1; NE: norepinephrine; NF-kB: nuclear factor-kappa B; PUFAs: poly-unsaturated fatty acids; SCFAs: short-chain fatty acids; TPH: thyrotropin-releasing hormone.

## References

[1] Kennedy S.H. (2008). Core symptoms of major depressive disorder: Relevance to diagnosis and treatment. Dialogues Clin. Neurosci..

[2] Frazier P., Liu Y., Asplund A., Meredith L., Nguyen-Feng V.N. (2021). US college student mental health and COVID-19: Comparing pre-pandemic and pandemic timepoints. J. Am. Coll. Health.

[3] Maes M., Galecki P., Chang Y.S., Berk M. (2011). A review on the oxidative and nitrosative stress (O&NS) pathways in major depression and their possible contribution to the (neuro)degenerative processes in that illness. Prog. Neuropsychopharmacol. Biol. Psychiatry.

[4] Moylan S., Berk M., Dean O.M., Samuni Y., Williams L.J., O’Neil A., Hayley A.C., Pasco J.A., Anderson G., Jacka F.N. (2014). Oxidative & nitrosative stress in depression: Why so much stress?. Neurosci. Biobehav. Rev..

[5] Salim S. (2014). Oxidative stress and psychological disorders. Curr. Neuropharmacol..

[6] Berthold-Losleben M., Himmerich H. (2008). The TNF-alpha system: Functional aspects in depression, narcolepsy and psychopharmacology. Curr. Neuropharmacol..

[7] Clapp M., Aurora N., Herrera L., Bhatia M., Wilen E., Wakefield S. (2017). Gut microbiota’s effect on mental health: The gut-brain axis. Clin. Pract..

[8] Foster J.A., McVey Neufeld K.A. (2013). Gut-brain axis: How the microbiome influences anxiety and depression. Trends Neurosci..

[9] Mello B.S., Monte A.S., McIntyre R.S., Soczynska J.K., Custodio C.S., Cordeiro R.C., Chaves J.H., Vasconcelos S.M., Nobre H.V., Florenco de Sousa F.C. (2013). Effects of doxycycline on depressive-like behavior in mice after lipopolysaccharide (LPS) administration. J. Psychiatr. Res..

[10] Miyaoka T., Wake R., Furuya M., Liaury K., Ieda M., Kawakami K., Tsuchie K., Taki M., Ishihara K., Araki T. (2012). Minocycline as adjunctive therapy for patients with unipolar psychotic depression: An open-label study. Prog. Neuropsychopharmacol. Biol. Psychiatry.

[11] Filipovic B.R., Filipovic B.F. (2014). Psychiatric comorbidity in the treatment of patients with inflammatory bowel disease. World J. Gastroenterol..

[12] Morshedi M., Valenlia K.B., Hosseinifard E.S., Shahabi P., Abbasi M.M., Ghorbani M., Barzegari A., Sadigh-Eteghad S., Saghafi-Asl M. (2018). Beneficial psychological effects of novel psychobiotics in diabetic rats: The interaction among the gut, blood and amygdala. J. Nutr. Biochem..

[13] Donati Zeppa S., Agostini D., Gervasi M., Annibalini G., Amatori S., Ferrini F., Sisti D., Piccoli G., Barbieri E., Sestili P. (2019). Mutual Interactions among Exercise, Sport Supplements and Microbiota. Nutrients.

[14] Cryan J.F., O’Riordan K.J., Cowan C.S.M., Sandhu K.V., Bastiaanssen T.F.S., Boehme M., Codagnone M.G., Cussotto S., Fulling C., Golubeva A.V. (2019). The Microbiota-Gut-Brain Axis. Physiol. Rev..

[15] Li J., Jia H., Cai X., Zhong H., Feng Q., Sunagawa S., Arumugam M., Kultima J.R., Prifti E., Nielsen T. (2014). An integrated catalog of reference genes in the human gut microbiome. Nat. Biotechnol..

[16] Godos J., Currenti W., Angelino D., Mena P., Castellano S., Caraci F., Galvano F., Del Rio D., Ferri R., Grosso G. (2020). Diet and Mental Health: Review of the Recent Updates on Molecular Mechanisms. Antioxidants.

[17] Rinninella E., Raoul P., Cintoni M., Franceschi F., Miggiano G.A.D., Gasbarrini A., Mele M.C. (2019). What is the Healthy Gut Microbiota Composition? A Changing Ecosystem across Age, Environment, Diet, and Diseases. Microorganisms.

[18] Fava F., Rizzetto L., Tuohy K.M. (2019). Gut microbiota and health: Connecting actors across the metabolic system. Proc. Nutr. Soc..

[19] Hills R.D., Pontefract B.A., Mishcon H.R., Black C.A., Sutton S.C., Theberge C.R. (2019). Gut Microbiome: Profound Implications for Diet and Disease. Nutrients.

[20] Dinan T.G., Cryan J.F. (2017). The Microbiome-Gut-Brain Axis in Health and Disease. Gastroenterol. Clin. N. Am..

[21] Simpson C.A., Diaz-Arteche C., Eliby D., Schwartz O.S., Simmons J.G., Cowan C.S.M. (2021). The gut microbiota in anxiety and depression—A systematic review. Clin. Psychol. Rev..

[22] Naseribafrouei A., Hestad K., Avershina E., Sekelja M., Linlokken A., Wilson R., Rudi K. (2014). Correlation between the human fecal microbiota and depression. Neurogastroenterol. Motil..

[23] Qu W., Liu S., Zhang W., Zhu H., Tao Q., Wang H., Yan H. (2019). Impact of traditional Chinese medicine treatment on chronic unpredictable mild stress-induced depression-like behaviors: Intestinal microbiota and gut microbiome function. Food Funct..

[24] Baj A., Moro E., Bistoletti M., Orlandi V., Crema F., Giaroni C. (2019). Glutamatergic Signaling along the Microbiota-Gut-Brain Axis. Int. J. Mol. Sci..

[25] Zheng P., Zeng B., Zhou C., Liu M., Fang Z., Xu X., Zeng L., Chen J., Fan S., Du X. (2016). Gut microbiome remodeling induces depressive-like behaviors through a pathway mediated by the host’s metabolism. Mol. Psychiatry.

[26] Jiang H., Ling Z., Zhang Y., Mao H., Ma Z., Yin Y., Wang W., Tang W., Tan Z., Shi J. (2015). Altered fecal microbiota composition in patients with major depressive disorder. Brain Behav. Immun..

[27] Dash S., Clarke G., Berk M., Jacka F.N. (2015). The gut microbiome and diet in psychiatry: Focus on depression. Curr. Opin. Psychiatry.

[28] Lach G., Schellekens H., Dinan T.G., Cryan J.F. (2018). Anxiety, Depression, and the Microbiome: A Role for Gut Peptides. Neurotherapeutics.

[29] Yang J., Zheng P., Li Y., Wu J., Tan X., Zhou J., Sun Z., Chen X., Zhang G., Zhang H. (2020). Landscapes of bacterial and metabolic signatures and their interaction in major depressive disorders. Sci. Adv..

[30] Osadchiy V., Martin C.R., Mayer E.A. (2019). The Gut-Brain Axis and the Microbiome: Mechanisms and Clinical Implications. Clin. Gastroenterol. Hepatol..

[31] O’Mahony S.M., Clarke G., Borre Y.E., Dinan T.G., Cryan J.F. (2015). Serotonin, tryptophan metabolism and the brain-gut-microbiome axis. Behav. Brain Res..

[32] Tuohy K.M., Venuti P., Cuva S., Furlanello C., Gasperotti M., Mancini A., Ceppa F., Cavalieri D., De Filippo C., Vrhovsek U., Tuohy K.M., Del Rio D. (2014). Diet and the Gut Microbiota—How the Gut: Brain Axis Impacts on Autism. Diet-Microbe Interactions in the Gut.

[33] Houghton D., Hardy T., Stewart C., Errington L., Day C.P., Trenell M.I., Avery L. (2018). Systematic review assessing the effectiveness of dietary intervention on gut microbiota in adults with type 2 diabetes. Diabetologia.

[34] Gershon M.D. (2013). 5-Hydroxytryptamine (serotonin) in the gastrointestinal tract. Curr. Opin. Endocrinol. Diabetes Obes..

[35] Liu Y., Wang H., Gui S., Zeng B., Pu J., Zheng P., Zeng L., Luo Y., Wu Y., Zhou C. (2021). Proteomics analysis of the gut-brain axis in a gut microbiota-dysbiosis model of depression. Transl. Psychiatry.

[36] Bosi A., Banfi D., Bistoletti M., Giaroni C., Baj A. (2020). Tryptophan Metabolites Along the Microbiota-Gut-Brain Axis: An Interkingdom Communication System Influencing the Gut in Health and Disease. Int. J. Tryptophan. Res..

[37] Donner N.C., Johnson P.L., Fitz S.D., Kellen K.E., Shekhar A., Lowry C.A. (2012). Elevated tph2 mRNA expression in a rat model of chronic anxiety. Depress. Anxiety.

[38] Kerckhoffs A.P., ter Linde J.J., Akkermans L.M., Samsom M. (2012). SERT and TPH-1 mRNA expression are reduced in irritable bowel syndrome patients regardless of visceral sensitivity state in large intestine. Am. J. Physiol. Gastrointest. Liver Physiol..

[39] Gao K., Mu C.L., Farzi A., Zhu W.Y. (2020). Tryptophan Metabolism: A Link Between the Gut Microbiota and Brain. Adv. Nutr..

[40] Williams B.B., Van Benschoten A.H., Cimermancic P., Donia M.S., Zimmermann M., Taketani M., Ishihara A., Kashyap P.C., Fraser J.S., Fischbach M.A. (2014). Discovery and characterization of gut microbiota decarboxylases that can produce the neurotransmitter tryptamine. Cell Host Microbe.

[41] Roager H.M., Licht T.R. (2018). Microbial tryptophan catabolites in health and disease. Nat. Commun..

[42] Behl T., Kaur I., Sehgal A., Singh S., Bhatia S., Al-Harrasi A., Zengin G., Bumbu A.G., Andronie-Cioara F.L., Nechifor A.C. (2021). The Footprint of Kynurenine Pathway in Neurodegeneration: Janus-Faced Role in Parkinson’s Disorder and Therapeutic Implications. Int. J. Mol. Sci..

[43] Kennedy P.J., Cryan J.F., Dinan T.G., Clarke G. (2017). Kynurenine pathway metabolism and the microbiota-gut-brain axis. Neuropharmacology.

[44] Martin-Gallausiaux C., Larraufie P., Jarry A., Beguet-Crespel F., Marinelli L., Ledue F., Reimann F., Blottiere H.M., Lapaque N. (2018). Butyrate Produced by Commensal Bacteria Down-Regulates Indolamine 2,3-Dioxygenase 1 (IDO-1) Expression via a Dual Mechanism in Human Intestinal Epithelial Cells. Front. Immunol..

[45] Jaglin M., Rhimi M., Philippe C., Pons N., Bruneau A., Goustard B., Dauge V., Maguin E., Naudon L., Rabot S. (2018). Indole, a Signaling Molecule Produced by the Gut Microbiota, Negatively Impacts Emotional Behaviors in Rats. Front. Neurosci..

[46] Rothhammer V., Mascanfroni I.D., Bunse L., Takenaka M.C., Kenison J.E., Mayo L., Chao C.C., Patel B., Yan R., Blain M. (2016). Type I interferons and microbial metabolites of tryptophan modulate astrocyte activity and central nervous system inflammation via the aryl hydrocarbon receptor. Nat. Med..

[47] Messaoud A., Mensi R., Douki W., Neffati F., Najjar M.F., Gobbi G., Valtorta F., Gaha L., Comai S. (2019). Reduced peripheral availability of tryptophan and increased activation of the kynurenine pathway and cortisol correlate with major depression and suicide. World J. Biol. Psychiatry.

[48] Liu H., Ding L., Zhang H., Mellor D., Wu H., Zhao D., Wu C., Lin Z., Yuan J., Peng D. (2018). The Metabolic Factor Kynurenic Acid of Kynurenine Pathway Predicts Major Depressive Disorder. Front. Psychiatry.

[49] Kelly J.R., Borre Y., O’Brien C., Patterson E., El Aidy S., Deane J., Kennedy P.J., Beers S., Scott K., Moloney G. (2016). Transferring the blues: Depression-associated gut microbiota induces neurobehavioural changes in the rat. J. Psychiatr. Res..

[50] Sies H. (2015). Oxidative stress: A concept in redox biology and medicine. Redox Biol..

[51] Barbieri E., Sestili P. (2012). Reactive oxygen species in skeletal muscle signaling. J. Signal Transduct..

[52] Cortie C.H., Hulbert A.J., Hancock S.E., Mitchell T.W., McAndrew D., Else P.L. (2015). Of mice, pigs and humans: An analysis of mitochondrial phospholipids from mammals with very different maximal lifespans. Exp. Gerontol..

[53] Calabrese E.J., Baldwin L.A. (2002). Defining hormesis. Hum. Exp. Toxicol..

[54] Cobley J.N., Fiorello M.L., Bailey D.M. (2018). 13 reasons why the brain is susceptible to oxidative stress. Redox Biol..

[55] Cortese-Krott M.M., Koning A., Kuhnle G.G.C., Nagy P., Bianco C.L., Pasch A., Wink D.A., Fukuto J.M., Jackson A.A., van Goor H. (2017). The Reactive Species Interactome: Evolutionary Emergence, Biological Significance, and Opportunities for Redox Metabolomics and Personalized Medicine. Antioxid. Redox Signal..

[56] da Rocha A.L., Pinto A.P., Kohama E.B., Pauli J.R., de Moura L.P., Cintra D.E., Ropelle E.R., da Silva A.S.R. (2019). The proinflammatory effects of chronic excessive exercise. Cytokine.

[57] Tapia P.C. (2006). Sublethal mitochondrial stress with an attendant stoichiometric augmentation of reactive oxygen species may precipitate many of the beneficial alterations in cellular physiology produced by caloric restriction, intermittent fasting, exercise and dietary phytonutrients: “Mitohormesis” for health and vitality. Med. Hypotheses.

[58] Mottawea W., Chiang C.K., Muhlbauer M., Starr A.E., Butcher J., Abujamel T., Deeke S.A., Brandel A., Zhou H., Shokralla S. (2016). Altered intestinal microbiota-host mitochondria crosstalk in new onset Crohn’s disease. Nat. Commun..

[59] Clark A., Mach N. (2017). The Crosstalk between the Gut Microbiota and Mitochondria during Exercise. Front. Physiol..

[60] Ribeiro M.F., Santos A.A., Afonso M.B., Rodrigues P.M., Sa Santos S., Castro R.E., Rodrigues C.M.P., Sola S. (2020). Diet-dependent gut microbiota impacts on adult neurogenesis through mitochondrial stress modulation. Brain Commun..

[61] Schluter J., Peled J.U., Taylor B.P., Markey K.A., Smith M., Taur Y., Niehus R., Staffas A., Dai A., Fontana E. (2020). The gut microbiota is associated with immune cell dynamics in humans. Nature.

[62] Stacy A., Andrade-Oliveira V., McCulloch J.A., Hild B., Oh J.H., Perez-Chaparro P.J., Sim C.K., Lim A.I., Link V.M., Enamorado M. (2021). Infection trains the host for microbiota-enhanced resistance to pathogens. Cell.

[63] Braniste V., Al-Asmakh M., Kowal C., Anuar F., Abbaspour A., Toth M., Korecka A., Bakocevic N., Ng L.G., Kundu P. (2014). The gut microbiota influences blood-brain barrier permeability in mice. Sci. Transl. Med..

[64] Marizzoni M., Cattaneo A., Mirabelli P., Festari C., Lopizzo N., Nicolosi V., Mombelli E., Mazzelli M., Luongo D., Naviglio D. (2020). Short-Chain Fatty Acids and Lipopolysaccharide as Mediators Between Gut Dysbiosis and Amyloid Pathology in Alzheimer’s Disease. J. Alzheimer’s Dis..

[65] Huie R.E., Padmaja S. (1993). The reaction of no with superoxide. Free Radic. Res. Commun..

[66] Tiso M., Schechter A.N. (2015). Nitrate reduction to nitrite, nitric oxide and ammonia by gut bacteria under physiological conditions. PLoS ONE.

[67] Leclerc M., Bedu-Ferrari C., Etienne-Mesmin L., Mariadassou M., Lebreuilly L., Tran S.L., Brazeau L., Mayeur C., Delmas J., Rue O. (2021). Nitric Oxide Impacts Human Gut Microbiota Diversity and Functionalities. mSystems.

[68] Saint-Georges-Chaumet Y., Edeas M. (2016). Microbiota-mitochondria inter-talk: Consequence for microbiota-host interaction. Pathog. Dis..

[69] Leschelle X., Goubern M., Andriamihaja M., Blottiere H.M., Couplan E., Gonzalez-Barroso M.D., Petit C., Pagniez A., Chaumontet C., Mignotte B. (2005). Adaptative metabolic response of human colonic epithelial cells to the adverse effects of the luminal compound sulfide. Biochim. Biophys. Acta.

[70] Fu Y., Ito M., Fujita Y., Ito M., Ichihara M., Masuda A., Suzuki Y., Maesawa S., Kajita Y., Hirayama M. (2009). Molecular hydrogen is protective against 6-hydroxydopamine-induced nigrostriatal degeneration in a rat model of Parkinson’s disease. Neurosci. Lett..

[71] Rose S., Bennuri S.C., Davis J.E., Wynne R., Slattery J.C., Tippett M., Delhey L., Melnyk S., Kahler S.G., MacFabe D.F. (2018). Butyrate enhances mitochondrial function during oxidative stress in cell lines from boys with autism. Transl. Psychiatry.

[72] Reese A.T., Cho E.H., Klitzman B., Nichols S.P., Wisniewski N.A., Villa M.M., Durand H.K., Jiang S., Midani F.S., Nimmagadda S.N. (2018). Antibiotic-induced changes in the microbiota disrupt redox dynamics in the gut. eLife.

[73] Teckentrup V., Neubert S., Santiago J.C.P., Hallschmid M., Walter M., Kroemer N.B. (2020). Non-invasive stimulation of vagal afferents reduces gastric frequency. Brain Stimul..

[74] Luca M., Luca A., Calandra C. (2013). Accelerated aging in major depression: The role of nitro-oxidative stress. Oxid. Med. Cell. Longev..

[75] Anderson G., Maes M. (2014). Oxidative/nitrosative stress and immuno-inflammatory pathways in depression: Treatment implications. Curr. Pharm. Des..

[76] Dobashi Y., Miyakawa Y., Yamamoto I., Amao H. (2011). Effects of intestinal microflora on superoxide dismutase activity in the mouse cecum. Exp. Anim..

[77] Szczesniak O., Hestad K.A., Hanssen J.F., Rudi K. (2016). Isovaleric acid in stool correlates with human depression. Nutr. Neurosci..

[78] Han L., Zhao L., Zhou Y., Yang C., Xiong T., Lu L., Deng Y., Luo W., Chen Y., Qiu Q. (2021). Altered metabolome and microbiome features provide clues in understanding irritable bowel syndrome and depression comorbidity. ISME J..

[79] Lai J., Li A., Jiang J., Yuan X., Zhang P., Xi C., Wu L., Wang Z., Chen J., Lu J. (2021). Metagenomic analysis reveals gut bacterial signatures for diagnosis and treatment outcome prediction in bipolar depression. Psychiatry Res..

[80] Meng C., Feng S., Hao Z., Dong C., Liu H. (2021). Antibiotics exposure attenuates chronic unpredictable mild stress-induced anxiety-like and depression-like behavior. Psychoneuroendocrinology.

[81] Gao X., Cao Q., Cheng Y., Zhao D., Wang Z., Yang H., Wu Q., You L., Wang Y., Lin Y. (2018). Chronic stress promotes colitis by disturbing the gut microbiota and triggering immune system response. Proc. Natl. Acad. Sci. USA.

[82] Martine P., Rebe C. (2019). Heat Shock Proteins and Inflammasomes. Int. J. Mol. Sci..

[83] Pasquali M.A., Harlow B.L., Soares C.N., Otto M.W., Cohen L.S., Minuzzi L., Gelain D.P., Moreira J.C.F., Frey B.N. (2018). A longitudinal study of neurotrophic, oxidative, and inflammatory markers in first-onset depression in midlife women. Eur. Arch. Psychiatry Clin. Neurosci..

[84] Liu H.Y., Roos S., Jonsson H., Ahl D., Dicksved J., Lindberg J.E., Lundh T. (2015). Effects of *Lactobacillus johnsonii* and *Lactobacillus reuteri* on gut barrier function and heat shock proteins in intestinal porcine epithelial cells. Physiol. Rep..

[85] Song J., Zhou N., Ma W., Gu X., Chen B., Zeng Y., Yang L., Zhou M. (2019). Modulation of gut microbiota by chlorogenic acid pretreatment on rats with adrenocorticotropic hormone induced depression-like behavior. Food Funct..

[86] Chi L., Khan I., Lin Z., Zhang J., Lee M.Y.S., Leong W., Hsiao W.L.W., Zheng Y. (2020). Fructo-oligosaccharides from *Morinda officinalis* remodeled gut microbiota and alleviated depression features in a stress rat model. Phytomedicine.

[87] Maes M., Kubera M., Leunis J.C., Berk M. (2012). Increased IgA and IgM responses against gut commensals in chronic depression: Further evidence for increased bacterial translocation or leaky gut. J. Affect. Disord..

[88] Ceppa F., Mancini A., Tuohy K. (2019). Current evidence linking diet to gut microbiota and brain development and function. Int. J. Food Sci. Nutr..

[89] Dinan T.G., Stanton C., Cryan J.F. (2013). Psychobiotics: A novel class of psychotropic. Biol. Psychiatry.

[90] Sarkar A., Lehto S.M., Harty S., Dinan T.G., Cryan J.F., Burnet P.W.J. (2016). Psychobiotics and the Manipulation of Bacteria-Gut-Brain Signals. Trends Neurosci..

[91] Sudo N., Chida Y., Aiba Y., Sonoda J., Oyama N., Yu X.N., Kubo C., Koga Y. (2004). Postnatal microbial colonization programs the hypothalamic-pituitary-adrenal system for stress response in mice. J. Physiol..

[92] Roshchina V.V. (2016). New Trends and Perspectives in the Evolution of Neurotransmitters in Microbial, Plant, and Animal Cells. Adv. Exp. Med. Biol..

[93] Yano J.M., Yu K., Donaldson G.P., Shastri G.G., Ann P., Ma L., Nagler C.R., Ismagilov R.F., Mazmanian S.K., Hsiao E.Y. (2015). Indigenous bacteria from the gut microbiota regulate host serotonin biosynthesis. Cell.

[94] Liu Y.W., Liu W.H., Wu C.C., Juan Y.C., Wu Y.C., Tsai H.P., Wang S., Tsai Y.C. (2016). Psychotropic effects of *Lactobacillus plantarum* PS128 in early life-stressed and naive adult mice. Brain Res..

[95] Liang S., Wang T., Hu X., Luo J., Li W., Wu X., Duan Y., Jin F. (2015). Administration of *Lactobacillus helveticus* NS8 improves behavioral, cognitive, and biochemical aberrations caused by chronic restraint stress. Neuroscience.

[96] Savignac H.M., Kiely B., Dinan T.G., Cryan J.F. (2014). Bifidobacteria exert strain-specific effects on stress-related behavior and physiology in BALB/c mice. Neurogastroenterol. Motil..

[97] Bravo J.A., Forsythe P., Chew M.V., Escaravage E., Savignac H.M., Dinan T.G., Bienenstock J., Cryan J.F. (2011). Ingestion of *Lactobacillus* strain regulates emotional behavior and central GABA receptor expression in a mouse via the vagus nerve. Proc. Natl. Acad. Sci. USA.

[98] Bercik P., Verdu E.F., Foster J.A., Macri J., Potter M., Huang X., Malinowski P., Jackson W., Blennerhassett P., Neufeld K.A. (2010). Chronic gastrointestinal inflammation induces anxiety-like behavior and alters central nervous system biochemistry in mice. Gastroenterology.

[99] Desbonnet L., Garrett L., Clarke G., Kiely B., Cryan J.F., Dinan T.G. (2010). Effects of the probiotic *Bifidobacterium infantis* in the maternal separation model of depression. Neuroscience.

[100] Wang H., He S., Xin J., Zhang T., Sun N., Li L., Ni X., Zeng D., Ma H., Bai Y. (2021). Psychoactive Effects of *Lactobacillus johnsonii* against Restraint Stress-Induced Memory Dysfunction in Mice through Modulating Intestinal Inflammation and permeability—A Study Based on the Gut-Brain Axis Hypothesis. Front. Pharmacol..

[101] Allen A.P., Hutch W., Borre Y.E., Kennedy P.J., Temko A., Boylan G., Murphy E., Cryan J.F., Dinan T.G., Clarke G. (2016). Bifidobacterium longum 1714 as a translational psychobiotic: Modulation of stress, electrophysiology and neurocognition in healthy volunteers. Transl. Psychiatry.

[102] Mohammadi A.A., Jazayeri S., Khosravi-Darani K., Solati Z., Mohammadpour N., Asemi Z., Adab Z., Djalali M., Tehrani-Doost M., Hosseini M. (2016). The effects of probiotics on mental health and hypothalamic-pituitary-adrenal axis: A randomized, double-blind, placebo-controlled trial in petrochemical workers. Nutr. Neurosci..

[103] Messaoudi M., Lalonde R., Violle N., Javelot H., Desor D., Nejdi A., Bisson J.F., Rougeot C., Pichelin M., Cazaubiel M. (2011). Assessment of psychotropic-like properties of a probiotic formulation (*Lactobacillus helveticus* R0052 and *Bifidobacterium longum* R0175) in rats and human subjects. Br. J. Nutr..

[104] Cheng L.H., Liu Y.W., Wu C.C., Wang S., Tsai Y.C. (2019). Psychobiotics in mental health, neurodegenerative and neurodevelopmental disorders. J. Food Drug Anal..

[105] Kazemi A., Noorbala A.A., Azam K., Eskandari M.H., Djafarian K. (2019). Effect of probiotic and prebiotic vs placebo on psychological outcomes in patients with major depressive disorder: A randomized clinical trial. Clin. Nutr..

[106] Burokas A., Arboleya S., Moloney R.D., Peterson V.L., Murphy K., Clarke G., Stanton C., Dinan T.G., Cryan J.F. (2017). Targeting the Microbiota-Gut-Brain Axis: Prebiotics Have Anxiolytic and Antidepressant-like Effects and Reverse the Impact of Chronic Stress in Mice. Biol. Psychiatry.

[107] Liu Y., Wu Z., Cheng L., Zhang X., Yang H. (2021). The role of the intestinal microbiota in the pathogenesis of host depression and mechanism of TPs relieving depression. Food Funct..

[108] Zhou N., Gu X., Zhuang T., Xu Y., Yang L., Zhou M. (2020). Gut Microbiota: A Pivotal Hub for Polyphenols as Antidepressants. J. Agric. Food Chem..

[109] Walsh C.J., Guinane C.M., O’Toole P.W., Cotter P.D. (2014). Beneficial modulation of the gut microbiota. FEBS Lett..

[110] Lavefve L., Howard L.R., Carbonero F. (2020). Berry polyphenols metabolism and impact on human gut microbiota and health. Food Funct..

[111] Liu X., Cao S., Zhang X. (2015). Modulation of Gut Microbiota-Brain Axis by Probiotics, Prebiotics, and Diet. J. Agric. Food Chem..

[112] Zhou J., Tang L., Shen C.L., Wang J.S. (2018). Green tea polyphenols modify gut-microbiota dependent metabolisms of energy, bile constituents and micronutrients in female Sprague-Dawley rats. J. Nutr. Biochem..

[113] Rothenberg D.O., Zhang L. (2019). Mechanisms Underlying the Anti-Depressive Effects of Regular Tea Consumption. Nutrients.

[114] Kelly J.R., Clarke G., Cryan J.F., Dinan T.G. (2016). Brain-gut-microbiota axis: Challenges for translation in psychiatry. Ann. Epidemiol..

[115] Kawabata K., Kawai Y., Terao J. (2010). Suppressive effect of quercetin on acute stress-induced hypothalamic-pituitary-adrenal axis response in Wistar rats. J. Nutr. Biochem..

[116] Ge J.F., Peng L., Cheng J.Q., Pan C.X., Tang J., Chen F.H., Li J. (2013). Antidepressant-like effect of resveratrol: Involvement of antioxidant effect and peripheral regulation on HPA axis. Pharmacol. Biochem. Behav..

[117] Yrondi A., Sporer M., Peran P., Schmitt L., Arbus C., Sauvaget A. (2018). Electroconvulsive therapy, depression, the immune system and inflammation: A systematic review. Brain Stimul..

[118] Karasawa K., Uzuhashi Y., Hirota M., Otani H. (2011). A matured fruit extract of date palm tree (*Phoenix dactylifera* L.) stimulates the cellular immune system in mice. J. Agric. Food Chem..

[119] Perez-Berezo T., Franch A., Castellote C., Castell M., Perez-Cano F.J. (2012). Mechanisms involved in down-regulation of intestinal IgA in rats by high cocoa intake. J. Nutr. Biochem..

[120] Cuevas A., Saavedra N., Salazar L.A., Abdalla D.S. (2013). Modulation of immune function by polyphenols: Possible contribution of epigenetic factors. Nutrients.

[121] Borutinskaite V., Virksaite A., Gudelyte G., Navakauskiene R. (2018). Green tea polyphenol EGCG causes anti-cancerous epigenetic modulations in acute promyelocytic leukemia cells. Leuk. Lymphoma.

[122] Inserra A., Rogers G.B., Licinio J., Wong M.L. (2018). The Microbiota-Inflammasome Hypothesis of Major Depression. BioEssays.

[123] Moreno-Indias I., Sanchez-Alcoholado L., Perez-Martinez P., Andres-Lacueva C., Cardona F., Tinahones F., Queipo-Ortuno M.I. (2016). Red wine polyphenols modulate fecal microbiota and reduce markers of the metabolic syndrome in obese patients. Food Funct..

[124] Barrett E., Ross R.P., O’Toole P.W., Fitzgerald G.F., Stanton C. (2012). Gamma-Aminobutyric acid production by culturable bacteria from the human intestine. J. Appl. Microbiol..

[125] Gonthier M.P., Verny M.A., Besson C., Remesy C., Scalbert A. (2003). Chlorogenic acid bioavailability largely depends on its metabolism by the gut microflora in rats. J. Nutr..

[126] Chen J., Lin D., Zhang C., Li G., Zhang N., Ruan L., Yan Q., Li J., Yu X., Xie X. (2015). Antidepressant-like effects of ferulic acid: Involvement of serotonergic and norepinergic systems. Metab. Brain Dis..

[127] Zeni A.L.B., Camargo A., Dalmagro A.P. (2017). Ferulic acid reverses depression-like behavior and oxidative stress induced by chronic corticosterone treatment in mice. Steroids.

[128] Takeda H., Tsuji M., Inazu M., Egashira T., Matsumiya T. (2002). Rosmarinic acid and caffeic acid produce antidepressive-like effect in the forced swimming test in mice. Eur. J. Pharmacol..

[129] Tomas-Barberan F.A., Selma M.V., Espin J.C. (2016). Interactions of gut microbiota with dietary polyphenols and consequences to human health. Curr. Opin. Clin. Nutr. Metab. Care.

[130] Parkar S.G., Trower T.M., Stevenson D.E. (2013). Fecal microbial metabolism of polyphenols and its effects on human gut microbiota. Anaerobe.

[131] Caspani G., Kennedy S., Foster J.A., Swann J. (2019). Gut microbial metabolites in depression: Understanding the biochemical mechanisms. Microb. Cell.

[132] Silva Y.P., Bernardi A., Frozza R.L. (2020). The Role of Short-Chain Fatty Acids From Gut Microbiota in Gut-Brain Communication. Front. Endocrinol..

[133] Skonieczna-Zydecka K., Grochans E., Maciejewska D., Szkup M., Schneider-Matyka D., Jurczak A., Loniewski I., Kaczmarczyk M., Marlicz W., Czerwinska-Rogowska M. (2018). Faecal Short Chain Fatty Acids Profile is Changed in Polish Depressive Women. Nutrients.

[134] Li J., Hou L., Wang C., Jia X., Qin X., Wu C. (2018). Short Term Intrarectal Administration of Sodium Propionate Induces Antidepressant-Like Effects in Rats Exposed to Chronic Unpredictable Mild Stress. Front. Psychiatry.

[135] Nankova B.B., Agarwal R., MacFabe D.F., La Gamma E.F. (2014). Enteric bacterial metabolites propionic and butyric acid modulate gene expression, including CREB-dependent catecholaminergic neurotransmission, in PC12 cells—Possible relevance to autism spectrum disorders. PLoS ONE.

[136] Graff J., Tsai L.H. (2013). The potential of HDAC inhibitors as cognitive enhancers. Annu. Rev. Pharmacol. Toxicol..

[137] Stilling R.M., van de Wouw M., Clarke G., Stanton C., Dinan T.G., Cryan J.F. (2016). The neuropharmacology of butyrate: The bread and butter of the microbiota-gut-brain axis?. Neurochem. Int..

[138] Appleton K.M., Sallis H.M., Perry R., Ness A.R., Churchill R. (2015). Omega-3 fatty acids for depression in adults. Cochrane Database Syst. Rev..

[139] Mocking R.J., Harmsen I., Assies J., Koeter M.W., Ruhe H.G., Schene A.H. (2016). Meta-analysis and meta-regression of omega-3 polyunsaturated fatty acid supplementation for major depressive disorder. Transl. Psychiatry.

[140] Mozaffari-Khosravi H., Yassini-Ardakani M., Karamati M., Shariati-Bafghi S.E. (2013). Eicosapentaenoic acid versus docosahexaenoic acid in mild-to-moderate depression: A randomized, double-blind, placebo-controlled trial. Eur. Neuropsychopharmacol..

[141] Mischoulon D., Best-Popescu C., Laposata M., Merens W., Murakami J.L., Wu S.L., Papakostas G.I., Dording C.M., Sonawalla S.B., Nierenberg A.A. (2008). A double-blind dose-finding pilot study of docosahexaenoic acid (DHA) for major depressive disorder. Eur. Neuropsychopharmacol..

[142] Malaspina A., Michael-Titus A.T. (2008). Is the modulation of retinoid and retinoid-associated signaling a future therapeutic strategy in neurological trauma and neurodegeneration?. J. Neurochem..

[143] Sherwin J.C., Reacher M.H., Dean W.H., Ngondi J. (2012). Epidemiology of vitamin A deficiency and xerophthalmia in at-risk populations. Trans. R. Soc. Trop. Med. Hyg..

[144] Chen B.W., Zhang K.W., Chen S.J., Yang C., Li P.G. (2021). Vitamin A Deficiency Exacerbates Gut Microbiota Dysbiosis and Cognitive Deficits in Amyloid Precursor Protein/Presenilin 1 Transgenic Mice. Front. Aging Neurosci..

[145] Lyu Y., Wu L., Wang F., Shen X., Lin D. (2018). Carotenoid supplementation and retinoic acid in immunoglobulin A regulation of the gut microbiota dysbiosis. Exp. Biol. Med..

[146] Elsherbiny N.M., Salama M.F., Said E., El-Sherbiny M., Al-Gayyar M.M. (2016). Crocin protects against doxorubicin-induced myocardial toxicity in rats through down-regulation of inflammatory and apoptic pathways. Chem. Biol. Interact..

[147] Hosseinzadeh H., Sadeghnia H.R., Ghaeni F.A., Motamedshariaty V.S., Mohajeri S.A. (2012). Effects of saffron (*Crocus sativus* L.) and its active constituent, crocin, on recognition and spatial memory after chronic cerebral hypoperfusion in rats. Phytother. Res..

[148] Shafiee M., Arekhi S., Omranzadeh A., Sahebkar A. (2018). Saffron in the treatment of depression, anxiety and other mental disorders: Current evidence and potential mechanisms of action. J. Affect. Disord..

[149] Vahdati Hassani F., Naseri V., Razavi B.M., Mehri S., Abnous K., Hosseinzadeh H. (2014). Antidepressant effects of crocin and its effects on transcript and protein levels of CREB, BDNF, and VGF in rat hippocampus. DARU J. Pharm. Sci..

[150] Xiao Q., Shu R., Wu C., Tong Y., Xiong Z., Zhou J., Yu C., Xie X., Fu Z. (2020). Crocin-I alleviates the depression-like behaviors probably via modulating “microbiota-gut-brain” axis in mice exposed to chronic restraint stress. J. Affect. Disord..

[151] Zhao B., Wu J., Li J., Bai Y., Luo Y., Ji B., Xia B., Liu Z., Tan X., Lv J. (2020). Lycopene Alleviates DSS-Induced Colitis and Behavioral Disorders via Mediating Microbes-Gut-Brain Axis Balance. J. Agric. Food Chem..

[152] Ge H., Yang T., Sun J., Zhang D. (2020). Associations between dietary carotenoid intakes and the risk of depressive symptoms. Food Nutr. Res..

[153] Kondo S., El Omri A., Han J., Isoda H. (2015). Antidepressant-like effects of rosmarinic acid through mitogen-activated protein kinase phosphatase-1 and brain-derived neurotrophic factor modulation. J. Funct. Foods.

[154] Girish C., Raj V., Arya J., Balakrishnan S. (2012). Evidence for the involvement of the monoaminergic system, but not the opioid system in the antidepressant-like activity of ellagic acid in mice. Eur. J. Pharmacol..

[155] Gujral S., Aizenstein H., Reynolds C.F., Butters M.A., Erickson K.I. (2017). Exercise effects on depression: Possible neural mechanisms. Gen. Hosp. Psychiatry.

[156] Ernst C., Olson A.K., Pinel J.P., Lam R.W., Christie B.R. (2006). Antidepressant effects of exercise: Evidence for an adult-neurogenesis hypothesis?. J. Psychiatry Neurosci..

[157] Dalton A., Mermier C., Zuhl M. (2019). Exercise influence on the microbiome-gut-brain axis. Gut Microbes.

[158] Sheng L., Wang Y., Jiang A., Zhou Y., Zhou H. (2021). Effect of Regular Physical Exercise on Gut Microbiota and Depressive Behaviors in Rats. J. Food Qual..

[159] Nay K., Smiles W.J., Kaiser J., McAloon L.M., Loh K., Galic S., Oakhill J.S., Gundlach A.L., Scott J.W. (2021). Molecular Mechanisms Underlying the Beneficial Effects of Exercise on Brain Function and Neurological Disorders. Int. J. Mol. Sci..

[160] Clarke S.F., Murphy E.F., O’Sullivan O., Lucey A.J., Humphreys M., Hogan A., Hayes P., O’Reilly M., Jeffery I.B., Wood-Martin R. (2014). Exercise and associated dietary extremes impact on gut microbial diversity. Gut.

[161] Mailing L.J., Allen J.M., Buford T.W., Fields C.J., Woods J.A. (2019). Exercise and the Gut Microbiome: A Review of the Evidence, Potential Mechanisms, and Implications for Human Health. Exerc. Sport Sci. Rev..

[162] Donati Zeppa S., Amatori S., Sisti D., Gervasi M., Agostini D., Piccoli G., Pazienza V., Gobbi P., Rocchi M.B.L., Sestili P. (2021). Nine weeks of high-intensity indoor cycling training induced changes in the microbiota composition in non-athlete healthy male college students. J. Int. Soc. Sports Nutr..

[163] Queipo-Ortuno M.I., Seoane L.M., Murri M., Pardo M., Gomez-Zumaquero J.M., Cardona F., Casanueva F., Tinahones F.J. (2013). Gut microbiota composition in male rat models under different nutritional status and physical activity and its association with serum leptin and ghrelin levels. PLoS ONE.

[164] Allen J.M., Mailing L.J., Niemiro G.M., Moore R., Cook M.D., White B.A., Holscher H.D., Woods J.A. (2018). Exercise Alters Gut Microbiota Composition and Function in Lean and Obese Humans. Med. Sci. Sports Exerc..

[165] McFadzean R. (2014). Exercise Can Help Modulate Human Gut Microbiota. Bachelor’s Thesis.

[166] Petersen L.M., Bautista E.J., Nguyen H., Hanson B.M., Chen L., Lek S.H., Sodergren E., Weinstock G.M. (2017). Community characteristics of the gut microbiomes of competitive cyclists. Microbiome.

[167] Clark A., Mach N. (2016). Exercise-induced stress behavior, gut-microbiota-brain axis and diet: A systematic review for athletes. J. Int. Soc. Sports Nutr..

[168] Claes S., Myint A.M., Domschke K., Del-Favero J., Entrich K., Engelborghs S., De Deyn P., Mueller N., Baune B., Rothermundt M. (2011). The kynurenine pathway in major depression: Haplotype analysis of three related functional candidate genes. Psychiatry Res..

